# No evidence for distinct types in the evolution of SARS-CoV-2

**DOI:** 10.1093/ve/veaa034

**Published:** 2020-05-14

**Authors:** Oscar A MacLean, Richard J Orton, Joshua B Singer, David L Robertson

**Affiliations:** MRC-University of Glasgow Centre for Virus Research (CVR), Garscube Campus, 464 Bearsden Road, Glasgow G61 1QH, UK

**Keywords:** COVID-19, SARS-CoV-2, adaptation

## Abstract

A recent study by Tang et al. claimed that two major types of severe acute respiratory syndrome-coronavirus-2 (CoV-2) had evolved in the ongoing CoV disease-2019 pandemic and that one of these types was more ‘aggressive’ than the other. Given the repercussions of these claims and the intense media coverage of these types of articles, we have examined in detail the data presented by Tang et al., and show that the major conclusions of that paper cannot be substantiated. Using examples from other viral outbreaks, we discuss the difficulty in demonstrating the existence or nature of a functional effect of a viral mutation, and we advise against overinterpretation of genomic data during the pandemic.

Following the report of a pneumonia outbreak in late December 2019 (WHO 2020), the first severe acute respiratory syndrome-coronavirus-2 (SARS-CoV-2) genome sequence was made publicly available on the 10 January 2020. Real time sequencing of viral genomes can help to understand the transmission history of pandemics and provide insights into how the pathogen is evolving (Gardy and Loman 2018). Additionally, dynamic nomenclature systems, as has been proposed for CoV disease-2019 (COVID-19; Rambaut et al. 2020), can be useful for tracking purposes.

Up to the 12 March 2020, 396 high-quality genomes of SARS-CoV-2 have been released, displaying in total 301 unique nonsynonymous substitutions that is, mutations associated with amino acid replacements (Fig. 1). These data have provided useful epidemiological insights into the history of the pandemic, for example, demonstrating multiple introductions into different geographical areas (Deng et al. 2020; Gudbjartsson et al. 2020). Using these genomes, the timing of the last common ancestor of the outbreak is estimated to be around late November 2019 (Rambaut 2020), with an exponential growth of infections since that date. Estimates of the virus’ evolutionary rate are centred around 8 × 10^−4^ substitutions per site per year (Rambaut 2020; Su et al. 2020), which is broadly in line with those estimated from SARS-CoV-1 and Middle East respiratory syndrome (MERS; Zhao et al. 2004; Dudas et al., 2018), and about a third of that estimated for influenza B (Virk et al. 2020).

**Figure 1.**
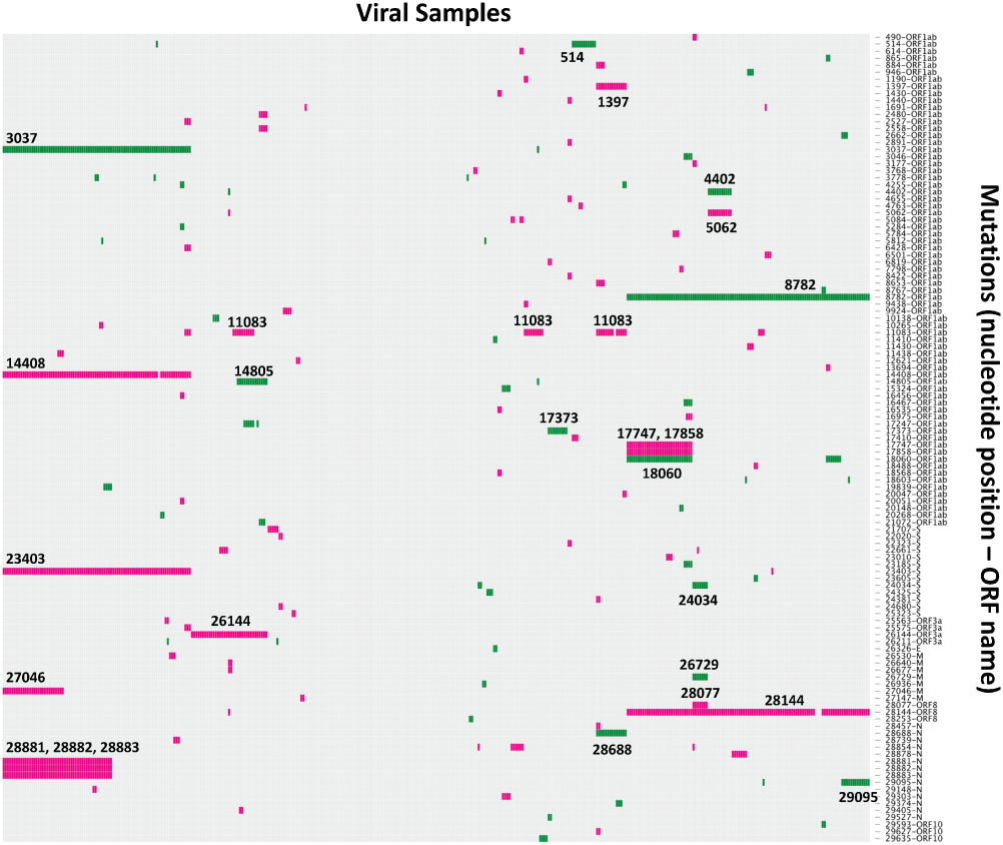
A visualization of the genetic variation observed in the SARS-CoV-2 pandemic sequences up until the 12 March 2020. Nonsynonymous (pink) and synonymous (green) substitutions (with respect to Wuhan-Hu-1, GenBank accession number MN908947) are represented in colour in each row, with rows labelled with the genome position and corresponding ORF on the side. The mutations are plotted in a grid format where each column is a sample and each row is a unique mutation at a given genome position; mutations have been filtered to only display those observed in more than one sample (seventy-four nonsynonymous and forty-one synonymous). The genome positions of some of the most common mutations have been labelled directly on the plot. The plot was created using the d3heatmap package in R, and the sample columns are clustered using Ward’s method.

An analysis of SARS-CoV-2 genetic data was published on the 3 March 2020 in the journal *National Science Review* by Tang et al. (2020). This study made two major claims that appear to have been reached by misinterpretation of the SARS-CoV-2 and the paper contains additional methodological limitations. We consider each claim in turn.

## 1. The first claim

The study proposes that there are two clearly definable ‘*major types*’ of SARS-CoV-2 in this outbreak and that they have differentiable transmission rates. Tang et al. term these two types ‘L’ and ‘S’:

two major types (L and S types): the S type is ancestral, and the L type evolved from S type. Intriguingly, the S and L types can be clearly defined by just two tightly linked SNPs at positions 8,782 (orf1ab: T8517C, synonymous) and 28,144 (ORF8: C251T, S84L).’

One nonsynonymous substitution, particularly one which has not been assessed for functional significance, is not sufficient to define a distinct ‘type’ nor ‘major type’. As of the 9 April 2020, there are 2,334 nonsynonymous substitutions that have been identified in the outbreak, catalogued in the CoV-GLUE resource at http://cov-glue.cvr.gla.ac.uk (Singer et al. 2018). At present, there is no evidence that any of these 2,334 point mutations have any significance in the functional context of within-host infections or transmission rates. Additionally, if one chooses to define ‘*types*’ purely on the basis of two mutations, it is unsurprising that these ‘*types*’ then differ by those two mutations.

However, Tang et al. further claim that these two types have differing transmission rates:

Thus far, we found that, although the L type is derived from the S type, L (∼70%) is more prevalent than S (∼30%) among the sequenced SARS-CoV-2 genomes we examined. This pattern suggests that L has a higher transmission rate than the S type.

The abstract of the paper goes even further, stating outright that: ‘*the S type, which is evolutionarily older and less aggressive…*’. It is, however, important to appreciate that finding a majority of samples with a particular mutation is not evidence that viruses with that mutation transmit more readily. To make this suggestion would, at the very minimum, require a comparison to be made to expectations under a null distribution assuming equal transmission rates. As this has not been performed by the authors, there is insufficient evidence to make this suggestion, and therefore it is incorrect (and, we would argue, irresponsible) to state that there is any difference in transmission rates. Genome sequence analysis alone is insufficient to demonstrate a functional effect of a mutation on virus phenotype, without assessing the probable impact of the amino acid replacement, and careful experimentation assessing any functional effect. Differences in the observed numbers of samples with and without this mutation are far more likely to be due to stochastic epidemiological effects and biased virus genome sampling among locations.

During a pandemic, as the virus spreads to new areas and countries that were previously uninfected, founder effects will have significant impacts on mutation frequencies. As a small number of virus copies spread into a local epidemic, any mutations present in the initial viral infections will rapidly become very common, even if they were rare in the particular geographical area which seeded the transmission. This is particularly likely to be the case in an outbreak caused by a novel virus such as SARS-CoV-2, as there are a large number of susceptible hosts for the virus, and numerous epidemics are being established around the world at different timepoints. These founder effects have also been observed in previous viral outbreaks for example, in chikungunya virus and multiple local HIV epidemics (Rambaut et al. 2001; Foley et al. 2000; Bhattacharya et al. 2007; Rai et al. 2010; Tsetsarkin et al. 2011).

Basic evolutionary theory predicts that selectively neutral mutations change in frequency over time through the process of genetic drift (Wright 1942). In a viral outbreak, each transmission event from one infected person to another is a random probabilistic event, with some infected individuals transmitting more or less often than others. Some infections may transmit at higher rates than others for a variety of reasons. These ‘super spreaders’ may have higher social contact rates or shed more virus for a longer period of time. These small-scale epidemiological phenomena add up over time to create substantial variation in the frequencies of mutations during an outbreak. It is also important to appreciate that the fewer infected hosts there are, the more these small-scale variations are likely to affect the frequency of mutations in the viral population. Given that the two mutations in question appear to have occurred very early on in the outbreak, when fewer individuals were infected, their frequency will very likely have been particularly influenced by genetic drift.

Any analysis of allele frequencies must also consider that the viral genomes which are sequenced are not a random sample of the global population, and are likely to be biased. In the SARS-CoV-2 pandemic, the sampling bias arises in two ways. First of all, the sampling of infections for sequencing is greatly biased by the country they occur in. For example, 80 per cent of confirmed COVID-19 cases up until 9 March 2020 came from China, but only 40 per cent of the SARS-CoV-2 full genome sequences derived from China. Second, as contact tracing is a significant driver of case detection, there will be a correlation between detected and sequenced samples, as they are often epidemiologically linked. This lack of independence between sampled genomes, in effect generates pseudoreplication of observed haplotypes. These factors combine to cause oversampling of particular genotypes and mutations, adding variance to the observed frequencies of mutations. This is likely to further exaggerate the variation in mutation frequencies driven by epidemiology, causing observed changes in mutation frequencies through time without any action of natural selection.

Examples from two previous viral outbreaks demonstrate these factors. A small number of mutations were observed to rise to high frequencies in both the Ebola and SARS-CoV-1 outbreaks. For both viruses, clearly demonstrating a functional effect of the mutations proved difficult, with some counterintuitive observations.

The A82V amino acid replacement in the GP protein from the 2013–16 Ebola outbreak illustrates the difficulty in demonstrating a functional effect of a mutation. Three new amino acid replacements in the Ebola outbreak rose in frequency to be found in >90 per cent of all sequenced genomes: R111C in the NP gene, A82V in the GP gene, and D759G in the L gene. The A82V replacement was of particular interest as it was located on the receptor binding interface. However, this rise in frequency alone was insufficient to make firm conclusions about the functional significance of this mutation. To demonstrate the significance of the A82V replacement, Diehl et al. (2016) performed numerous additional analyses. These included: predicting the structural impact of the change on the protein in silico; modelling the effect of the mutation on case fatality rate, controlling for viral loads, geographic location and access to healthcare; and in vitro experimental infection of three different human, and nine different nonhuman cell lines using viruses with and without this mutation. Despite finding significant evidence that virus infections with the A82V replacement showed higher mortality rates, and that the mutation enhanced infectivity of human and primate cell lines, the authors were not able to conclude that this mutation contributed to greater transmission and severity of the outbreak:

It is difficult to draw any conclusion about this hypothesis, though, since the frequency increase can also be attributed to stochastic effects, including founder effects as EBOV moved from Guinea into Sierra Leone and multiple re-introductions of GP-A82V back into Guinea.

At the same time, Urbanowicz et al. (2016) also found that A82V increased infectivity of human cell lines and decreased infectivity of bat cell lines ‘*supporting the hypothesis that A82V is a fitness adaptation*’. However, a follow-up study failed to find evidence of the mutation conferring higher viral titres or shedding rates in experimental infection of macaques (Marzi et al. 2018). The reason for this discrepancy between live animal models and cell lines is not yet understood, which means the functional significance of the A82V replacement remains unresolved.

A similar example can be found in the SARS-CoV-1 outbreak. In the initial phases of the outbreak, a 29 nucleotide (nt) deletion within open reading frame 8 (ORF8; the same ORF as the S84L replacement that was used to define S and L types in SARS-CoV-2) was identified, and viruses with this deletion subsequently became dominant within the outbreak (The Chinese SARS Molecular Epidemiology Consortium 2004). This mutation caused the splitting of ORF8 into two ORFs: ORF8a and ORF8b. It was hypothesized that this deletion was either neutral, with ORF8 being functionally unimportant (The Chinese SARS Molecular Epidemiology Consortium 2004), or that that it was adaptive, facilitating the spread of SARS-CoV-1 in humans (e.g. Chen et al. 2007; Wong et al. 2018). However, experimental infection of one bat and two human cell lines showed that the 29 nt deletion significantly reduced the replicative capability of SARS-CoV-1 (Muth et al. 2018). Additionally, deletion of the full ORF8 gene caused an even greater reduction in replicative capability. The spread of this apparently strongly deleterious mutation was hypothesized to be the result of a founder effect in the early period of the epidemic (Muth et al. 2018).

Combined, these factors and examples demonstrate that the frequency of a particular mutation in and of itself is not demonstrative of any functional significance.

## 2. The second claim

**Figure 2.**
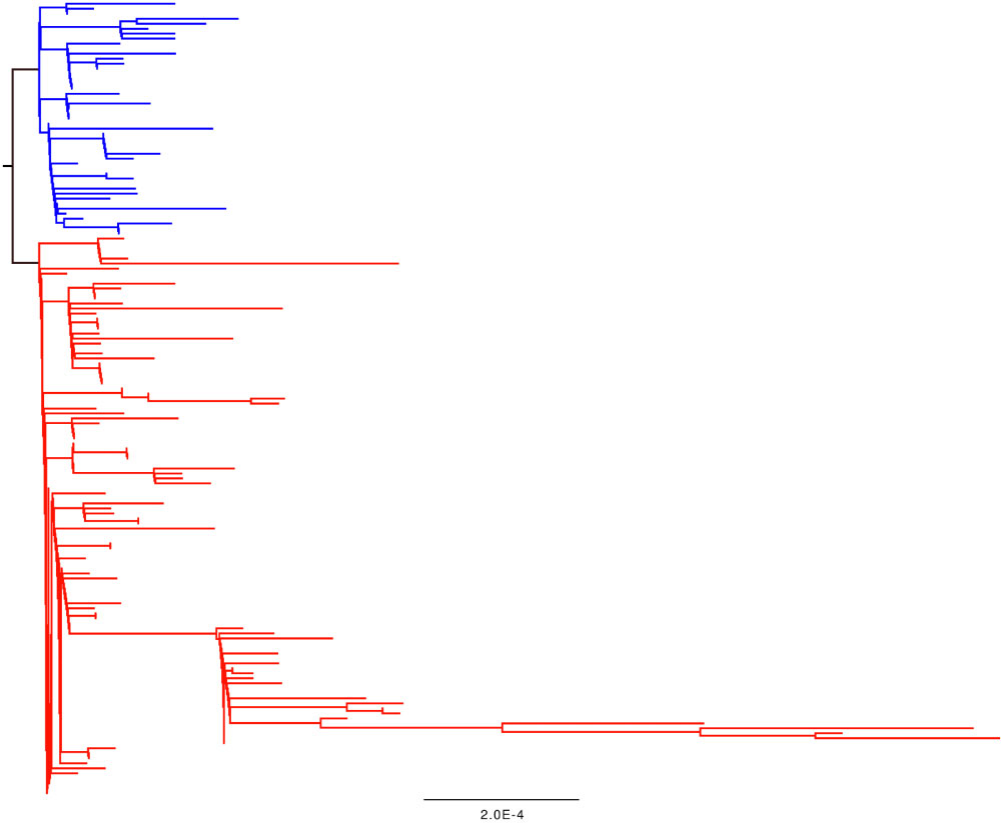
A phylogenetic tree of the SARS-CoV-2 outbreak data as of 2 March 2020. The tree was generated by the CoV-GLUE resource which uses the RAXML software (Stamatakis 2014). Branches and tips coloured blue have a serine at Codon 84 in ORF8, red tips and branches have a leucine.

Tang et al. (2020) compare the frequencies of nonsynonymous and synonymous substitutions in the data, claiming that there is significant evidence of selection suppressing the frequency of nonsynonymous substitutions in the outbreak. This analysis is flawed on three grounds:

First, the numbers in this figure do not make sense. According to the presented data, seven (synonymous) substitutions have a derived frequency of >50 per cent, and four of these mutations have derived frequencies >95 per cent in the population. A cursory glance at the tree in Fig. 2 shows that this cannot be true. ‘*Derived*’ in this context should mean a sequence change away from the genome of the last common ancestor of the outbreak. For four mutations to have derived frequencies >95 per cent, there would need to be a small number of samples which branch as a sister lineage to the rest of the outbreak tree. However, this is not the case.

**Figure 3.**
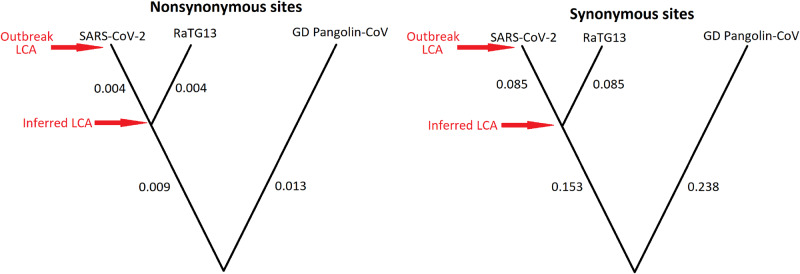
Schematic phylogenetic trees, not drawn to scale, inferred from nonsynonymous (left) and synonymous sites (right) using the estimated divergence values per site from Table 1 of Tang et al. (2020), assuming clock-like mutation rates. The last common ancestor (LCA) of the SARS-CoV-2 outbreak is much closer to that of the LCA shared with the bat-infecting RaTG13 sample in nonsynonymous sites than in synonymous sites. Accession numbers from GISAID for the RaTG13 and Guandong (GD) Pangolin-CoV samples are EPI_ISL_402131 and EPI_ISL_410721, respectively.

The only way Tang et al. can get the results they present is by defining the ancestral state not as the last common ancestor of the outbreak, but as the most recent common ancestor of the outbreak and the nearest bat sarbecovirus RaTG13. The most recent common ancestor of SARS-CoV-2 and RaTG13 existed many decades ago (Boni et al. 2020). As such, many mutations separate these two inferred ancestral states, especially at synonymous sites (Fig. 3).

Tang et al. estimate the ancestral state for each mutation independently, ignoring the very informative phylogenetic tree of the current outbreak, and the temporal information associated with each sample. This method only makes sense when using a much more closely related outgroup species to infer the ancestral states of mutations in a freely recombinant species with unlinked mutations with independent ancestry. Additionally, such methods should incorporate the inherent uncertainty in inferring the ancestral state (e.g. est-sfs; Keightley and Jackson 2018), which the implementation in Tang et al. does not.

Implementing this flawed method of inferring ancestral states in a viral outbreak context, where we assume there is no recombination, means that ‘*high-frequency derived mutations*’ are actually just new mutations in the outbreak that have mutated back to the inferred ancestral state. This generates an evolutionarily meaningless definition of ‘*derived*’ mutations.

Tang et al. claim seven synonymous and one nonsynonymous substitutions have a derived frequency >0.5. However, because synonymous sites are twenty times more diverged than nonsynonymous sites to the most closely related bat sequence RaTG13 (Figure 3), the difference between these two ancestral states is much larger in synonymous sites. Therefore, synonymous substitutions are much more likely to be mispolarized than nonsynonymous ones. This is because new synonymous substitutions in the outbreak are much more likely to mutate back to this deeper ancestral state in the tree than new nonsynonymous substitutions. Therefore, using this flawed definition of ‘*derived*’, an artefactual excess of high-frequency synonymous substitutions resembling purifying selection will be observed, without any such selection having occurred.

In addition, the way these data are presented in Tang et al.’s Fig. 2 will falsely suggest that purifying selection is acting, even if their methodology was sensible, and there were no such selection. The height of the bars in their figure compares the raw numbers of mutations at each frequency without scaling the heights of the bars for the number of each class of mutation. Because there is a greater number of nonsynonymous substitutions than synonymous substitutions in the population, and as most substitutions are expected to be at low frequency in a population regardless of the action of natural selection (Fay and Wu 2000), this presentation will always make it look like there’s proportionately more low-frequency nonsynonymous substitutions.

When interpreting their results, Tang et al. do not consider that sequencing error could be a driver of a relative excess of singleton nonsynonymous substitutions. This possibility is important because sequencing errors will be at low frequency as they are rare and cannot be transmitted, but real mutations can be at any frequency because they can be transmitted. Additionally, purifying selection can only act on real mutations, and not sequencing errors, so strongly deleterious/lethal nonsynonymous substitutions which cannot be observed as real mutations may appear as sequencing errors. Therefore, it is very possible that sequencing error mutations will have a higher nonsynonymous to synonymous ratio, and these mutations will be at low frequency. This pattern will mimic the action of purifying selection on circulating variation, suppressing the frequency of nonsynonymous substitutions.

On a more technical point, Tang et al. used the software PAML (Yang 2007) to estimate selection parameters and look for evidence of positive selection in the divergence between SARS-CoV-2 and other related CoVs. PAML does not allow for synonymous rate variation, but they explicitly state in the paper they believe there are mutational hotspots. Recent work has shown that false positive rates of positive selection inference are unacceptably high when such synonymous rate variation occurs (Wisotsky et al. 2020). Therefore, if there truly is synonymous rate variation, to reliably identify signatures of positive selection within the phylogeny of SARS-CoV-2, methods which model mutation rate variation must be used (e.g. provided by many of the models from the Hyphy package- Pond and Muse 2005).

Given the flaws described above, we believe that Tang et al.’s claims are clearly unsubstantiated. The widespread media interest in this article (186 articles at last count), and many comments on social media, suggests that the claim of increased aggressiveness in SARS-CoV-2 has already caused unnecessary concern and confusion at a crucial time in the pandemic.

A recent paper has proposed three ‘types’ of SARS-CoV-2 (Forster et al. 2020) and has also received substantial attention in the media. Although that paper does not make any claims of any functional differences among these ‘types’, many of the issues discussed above apply again to this work. The network Forster et al. produce uses the RaTG13 bat sarebecovirus sample to infer the ancestral state of the outbreak. By ignoring the temporal information given by the viral tree and the decades of evolution separating RaTG13 and SARS-CoV-2, the inferred ancestor of the outbreak in this network is likely to be incorrect. The choice of which and how many clusters in the network were named was made on the basis of the number of samples belonging to, and surrounding, each node. This methodology means that the sampling biases described earlier are very likely to be driving this classification.

Although rapid publication is critical for unfolding disease outbreaks, thorough and independent peer review should not be bypassed to get results published quickly. The current intensity of media interest in virology is unprecedented, and whilst rapid open-access research is paramount, researchers must be cautious of overinterpretation of data and the language used to describe results.
